# Acute morphine induces matrix metalloproteinase-9 up-regulation in primary sensory neurons to mask opioid-induced analgesia in mice

**DOI:** 10.1186/1744-8069-8-19

**Published:** 2012-03-25

**Authors:** Yen-Chin Liu, Temugin Berta, Tong Liu, Ping-Heng Tan, Ru-Rong Ji

**Affiliations:** 1 Sensory Plasticity Laboratory, Pain Research Center, Department of Anesthesiology, Brigham and Women's Hospital, Harvard Medical School, Boston, Massachusetts 02115, USA; 2 Department of Anesthesiology, College of Medicine, National Cheng Kung University, Tainan city, Taiwan; 3 Departments of Anesthesiology and Biomedical Engineering, E-DA Hospital/I-Shou University, Kaohsiung, Taiwan

**Keywords:** Dorsal root ganglion, Metalloprotease, MMP-9, mu opioid receptor (MOR), Opioid-induced analgesia, Opioid-induced hyperalgesia (OIH), Spinal cord

## Abstract

**Background:**

Despite decades of intense research efforts, actions of acute opioids are not fully understood. Increasing evidence suggests that in addition to well-documented antinociceptive effects opioids also produce paradoxical hyperalgesic and excitatory effects on neurons. However, most studies focus on the pronociceptive actions of chronic opioid exposure. Matrix metalloproteinase 9 (MMP-9) plays an important role in neuroinflammation and neuropathic pain development. We examined MMP-9 expression and localization in dorsal root ganglia (DRGs) after acute morphine treatment and, furthermore, the role of MMP-9 in modulating acute morphine-induced analgesia and hyperalgesia in mice.

**Results:**

Subcutaneous morphine induced a marked up-regulation of MMP-9 protein in DRGs but not spinal cords. Morphine also increased MMP-9 activity and mRNA expression in DRGs. MMP-9 up-regulation peaked at 2 h but returned to the baseline after 24 h. In DRG tissue sections, MMP-9 is expressed in small and medium-sized neurons that co-express mu opioid receptors (MOR). In DRG cultures, MOR agonists morphine, DAMGO, and remifentanil each increased MMP-9 expression in neurons, whereas the opioid receptor antagonist naloxone and the MOR-selective antagonist D-Phe-Cys-Tyr-D-Trp-Arg-Thr-Pen-Thr-NH_2 _(CTAP) suppressed morphine-induced MMP-9 expression. Notably, subcutaneous morphine-induced analgesia was enhanced and prolonged in *Mmp9 *knockout mice and also potentiated in wild-type mice receiving intrathecal injection of MMP-9 inhibitors. Consistently, intrathecal injection of specific siRNA targeting MMP-9 reduced MMP-9 expression in DRGs and enhanced and prolonged morphine analgesia. Subcutaneous morphine also produced heat hyperalgesia at 24 h, but this opioid-induced hyperalgesia was not enhanced after MMP-9 deletion or inhibition.

**Conclusions:**

Transient MMP-9 up-regulation in DRG neurons can mask opioid analgesia, without modulating opioid-induced hyperalgesia. Distinct molecular mechanisms (MMP-9 dependent and independent) control acute opioid-induced pronociceptive actions (anti-analgesia in the first several hours and hyperalgesia after 24 h). Targeting MMP-9 may improve acute opioid analgesia.

## Background

Opioids especially mu opioid receptor (MOR) agonists remain to be the most effective treatment for moderate to severe pain. MOR is expressed by primary sensory neurons including small-sized (C-fiber) and medium-sized (Aδ-fiber) neurons in the dorsal root ganglia (DRGs) [1-5]. MOR is also expressed in primary afferent terminals and lamina II interneurons in the spinal cord [1,6-8]. MOR agonist, such as [D-Ala2, N-Me-Phe4, Gly5-ol]-enkephalin (DAMGO) elicits potent presynaptic inhibition via suppressing N-type Ca^2+ ^channels and neutrotransmitter release in the superficial dorsal horn [9-12]. Postsynaptically, MOR agonists open G protein-coupled inwardly rectifying potassium (GIRK) channels and induce membrane hyperpolarization of dorsal horn neurons [12,13]. In addition, opioid produced by immune cells can also elicit peripheral analgesia by activating opioid receptors on nerve terminals [14,15].

Accumulating evidence from animal and human studies suggests that opioids also produce paradoxical excitatory and hyperalgesic effects. This phenomenon is known as opioid-induced hyperalgesia (OIH), as a result of up-regulation of pronociceptive pathways in the central and peripheral nervous systems [16-18]. A brief exposure to fentanyl or morphine induces long-lasting hyperalgesia [19,20]. An early study by Chen and Huang demonstrated that MOR agonist caused a sustained potentiation of NMDA receptor-mediated glutamate responses in spinal trigeminal neurons [21]. Recently, Drdla et al. (2009) reported that abrupt withdrawal from DAMGO induced long-term potentiation (LTP) in the spinal cord via NMDA receptor-mediated postsynaptic mechanisms [22,23]. Moreover, Zhou et al. (2010) demonstrated that DAMGO-induced LTP required presynaptic mechanisms and TRPV1-expressing primary afferents in the spinal cord [24].

Matrix metalloproteases (MMPs) consist of a large family of endopeptidases that require Zn^2^+ for their enzyme activity and play a critical role in inflammation through the cleavage of the extracellular matrix proteins, cytokines, and chemokines [25-27]. The gelatinases MMP-9 and MMP-2 are two of the best-studied MMP family members. The activity of MMP-9 is regulated by endogenous inhibitors, especially TIMP-1 (tissue inhibitor of metalloprotease-1) [28]. MMP-9 is involved in a wide range of CNS diseases including Alzheimer's, amyotrophic lateral sclerosis, multiple sclerosis, brain and spinal cord trauma, epilepsy, and stroke [29]. By degrading extracellular matrix, MMP-9 damages the blood-brain barrier, resulting in edema and vascular leakage in the CNS. Recently, we have demonstrated distinct roles of MMP-9 and MMP-2 in neuropathic pain development: (i) transient MMP-9 up-regulation after nerve injury is critical for the early-phase development of neuropathic pain; (ii) sustained MMP-2 up-regulation maintains neuropathic pain; and (iii) MMP-9 and MMP-2 induce the active cleavage of IL-1β (activation) in early and late-phase of nerve injury, respectively [28,30]. MMP-9 up-regulation in the spinal cord has also been implicated in chronic opioid-induced withdrawal syndrome (morphine dependence) through possible neuronal activation and interaction with NMDA receptors (NR1 and NR2B) via integrin-beta1 and NO pathways [31]. It is unclear whether MMP-9 also plays a role in acute opioid-induced pronociceptive responses. Our data demonstrated that acute morphine treatment via either subcutaneous or intrathecal route induced rapid and transient MMP-9 up-regulation (1-3 h) in primary sensory neurons to counteract opioid analgesia in the first several hours. In contrast, OIH at 24 h was not affected by MMP-9 deletion or inhibition.

## Results

### Subcutaneous morphine treatment increases MMP-9 expression and activity in DRGs

**Figure 1:**
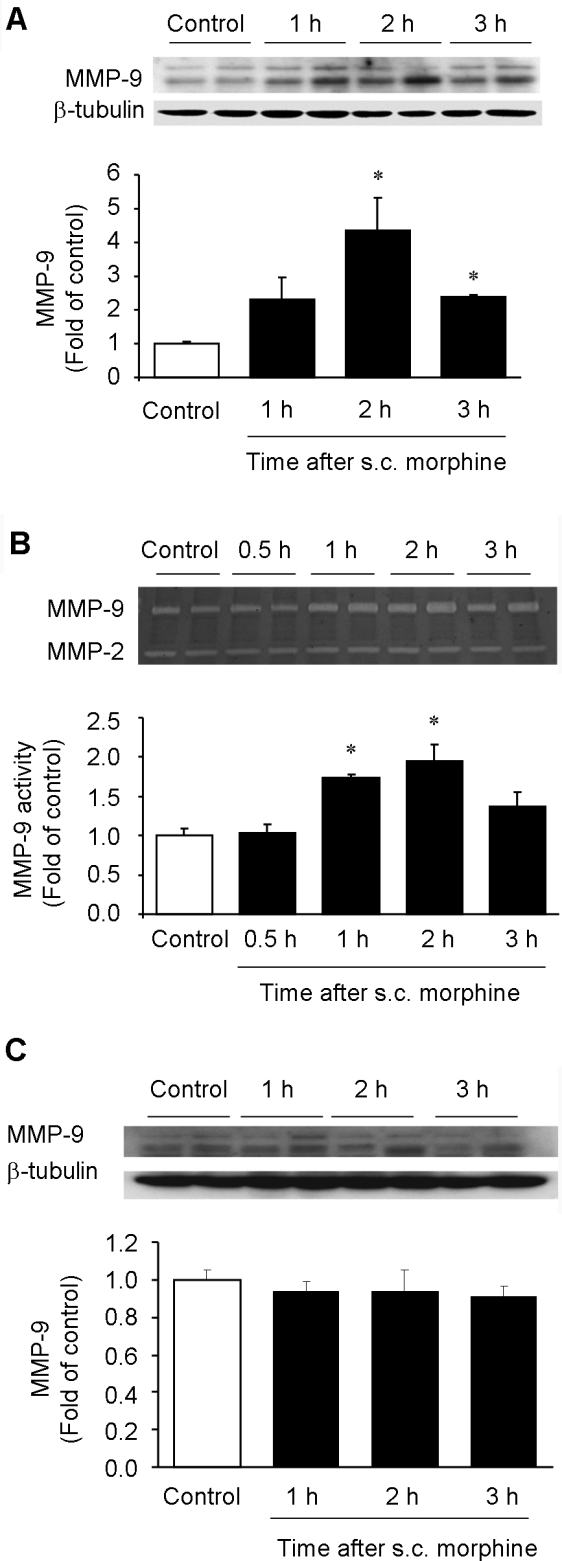
**Subcutaneous morphine increases MMP-9 expression and activity in DRGs**. **(A) **Western blotting showing the time course of MMP-9 expression in lumbar DRGs after morphine (s.c., 10 mg/kg). **(B) **Gelatin zymography showing the time course of MMP-9 activity in lumbar DRGs after morphine. Note that MMP-2 activity remains unchanged after morphine injection. **(C) **Western blotting showing the time course of MMP-9 expression in lumbar spinal cord dorsal horns after morphine (s.c., 10 mg/kg). Note that subcutaneous morphine only induces MMP-9 expression in DRGs but not spinal cords. *P < 0.05, compared to naive control, ANOVA followed by Bonferroni post hoc test, n = 4 mice.

As a first step to define the role of MMP-9 in opioid analgesia, we conducted a time course study to examine MMP-9 protein and activity levels in lumbar DRGs using Western blot and zymography, respectively. Western blotting revealed that MMP-9 began to increase at 1 h, peaked at 2 h (4.3 fold of control, *P *< 0.05, n = 4), and declined at 3 h (2.4 fold of control, *P *< 0.05, n = 4) (Figure 1A). Gelatin zymography showed a parallel increase of MMP-9 activity in the DRG at 1 h (1.71 fold of control, *P *< 0.05, n = 4) and 2 h (2.0 fold of control; *P *< 0.05, n = 4) (Figure 1B). Therefore, both the protein and activity levels of MMP-9 are increased in DRGs after morphine injection, peaking at 2 h. Notably, the activity of another gelatinase, MMP-2, a close family member of MMP-9, did not change after subcutaneous morphine (Figure 1B, *P *> 0.05, n = 4), suggesting a unique role of MMP-9 in response to acute opioid.

We also examined MMP-9 expression in the lumbar spinal cord of morphine-treated mice. In sharp contrast, we did not find significant changes in MMP-9 protein expression in the spinal cord dorsal horn at all the times we tested after morphine injection (Figure 1C, *P *> 0.05, n = 4). Neither did we observe MMP-9 activity change in the dorsal horn 2 h after the morphine treatment (Additional file 1).

**Figure 2:**
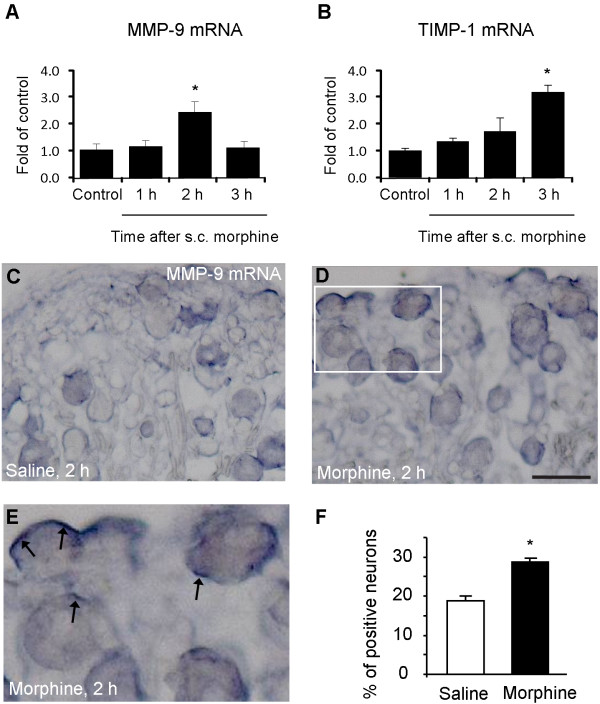
**Subcutaneous morphine induces MMP-9 and TIMP-1 mRNA up-regulation in DRGs**. **(A, B) **RT-PCR showing time course of subcutaneous morphine-induced MMP-9 (A) and TIMP-1 (B) mRNA expression in DRGs. *P < 0.05, compared to naive control, ANOVA followed by Bonferroni post hoc test, n = 4 mice. **(C-E) ***In situ *hybridization showing MMP-9 mRNA expression in DRG neurons 2 h after subcutaneous injection of saline (A) and morphine (B, C). Scale, 50 μm. The box in D is enlarged in E. Note strong MMP-9 mRNA staining (arrows) near cell surface following morphine treatment. **(F) **Percentage of MMP-9 mRNA-positive neurons in DRGs. *P < 0.05, compared to vehicle (saline), Student's t-test, n = 4 mice.

To determine whether MMP-9 up-regulation is caused by transcriptional regulation, we also performed quantitative RT-PCR to determine MMP-9 and TIMP-1 mRNA levels, in DRGs at different times following acute morphine treatment. Morphine elicited a significant increase in MMP-9 mRNA expression at 2 h (2.3 fold of control, *P *< 0.05, n = 4, Figure 2A) but a delayed increase in TIMP-1 mRNA expression at 3 h (3.2 fold of control, *P *< 0.05, n = 4, Figure 2B). Up-regulation of TIMP-1, the endogenous inhibitor of MMP-9, is likely to suppress MMP-9 expression to control its detrimental actions. By contrast, morphine did not alter MMP-9 mRNA expression in the spinal cord dorsal horn at all the times we tested (Additional file 1).

*In situ *hybridization confirmed a significant increase of MMP-9 mRNA levels in lumbar DRGs 2 h after morphine injection (*P *< 0.05, n = 4, Figure 2C-F). MMP-9 mRNA expression was only observed in DRG neurons (Figure 2C-E). Interestingly, after morphine treatment, strong mRNA signals were found in subcellular regions close to the surface of DRG neurons (Figure 2D, E).

### Subcutaneous morphine induces MMP-9 in MOR-expressing DRG neurons

**Figure 3:**
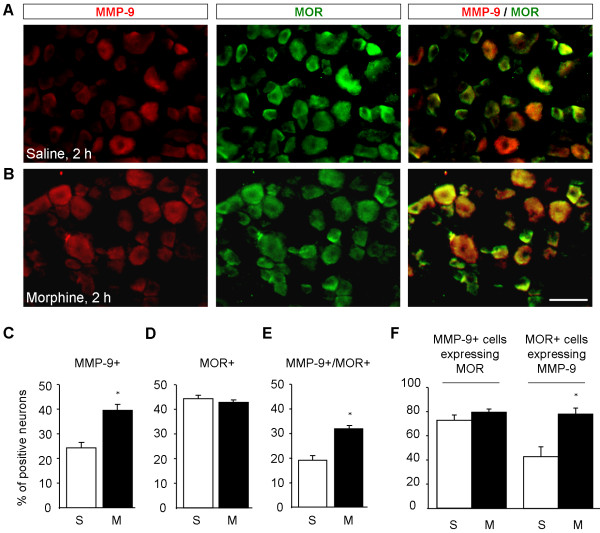
**Subcutaneous morphine-induced MMP-9 increase is enriched in mu opioid receptor (MOR)-expressing DRG neurons**. **(A, B) **Double staining showing colocalization of MMP-9 and MOR in DRG neurons of saline (A) and morphine (B) treated mice (s.c., 10 mg/kg). Scale, 50 μm. **(C, D) **Percentage of MMP-9+ (C) and MOR + (D) neurons in DRGs 2 h after saline and morphine injection. **(E) **Percentage of MMP-9 and MOR double-positive neurons in DRGs 2 h after saline and morphine injection. **(F) **Percentage of MMP-9-positive neurons expressing MOR and percentage of MOR-positive neurons expressing MMP-9 in DRGs 2 h after saline and morphine injection. S, saline; M, morphine. *P < 0.05, compared to saline, Student's t-test, n = 4 mice.

**Figure 4:**
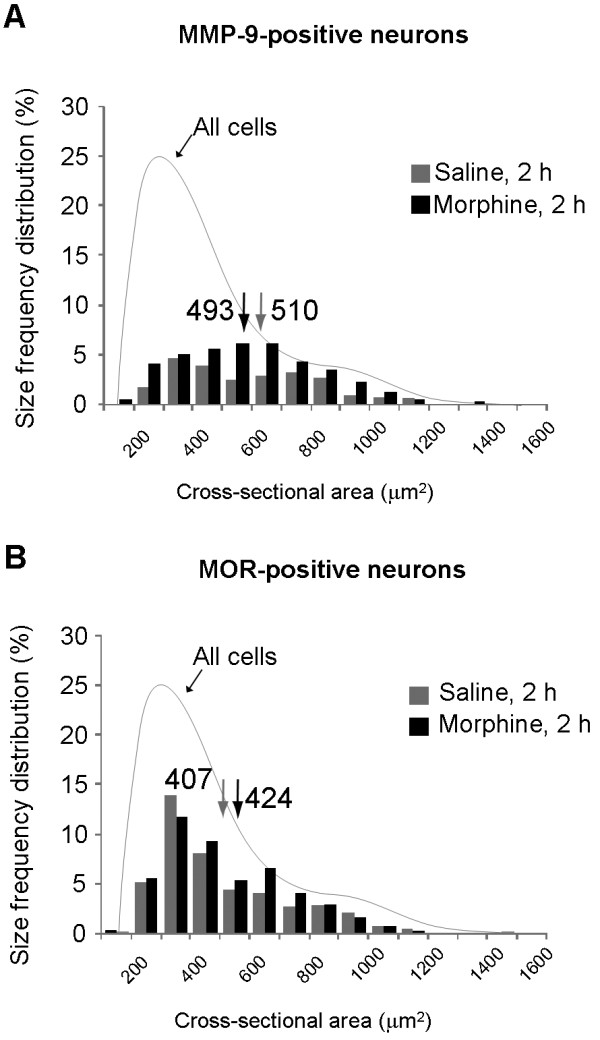
**Actue morphine treatment does not change the sizes of MMP-9- and MOR-positive neurons in DRGs**. **(A, B) **Size frequency distribution (percentage) of MMP-9-positive neurons (A) and MOR-positive neurons (B) in DRGs 2 h after subcutaneous saline and morphine. 300-500 neurons from 3 animals were plotted in each group. Note that MMP-9 and MOR are mainly expressed in small- and medium-sized neurons.

Immunohistochemistry showed that MMP-9 was expressed in 24.2 ± 3.9% DRG neurons in saline-treated mice, and this percentage was significantly increased 2 h after morphine treatment (*P *< 0.05, n = 4, Figure 3A-C), in support of the Western blot results. MMP-9 immunoreactivity was abolished in *Mmp9 *knockout mice or after omitting the primary antibody (Additional file 1). Of note, MOR expression per se did not change at 2 h after morphine injection (Figure 3A, B, and 3D). Double staining indicated that the percentage of DRG neurons expressing both MMP-9 and MOR increased from 18.9 ± 3.9% in vehicle-treated animals to 31.7 ± 3.6% in morphine-treated animals (1.7 fold of control, *P *< 0.05, n = 4) (Figure 3E). Further characterization demonstrated that MMP-9 was largely colocalized with MOR and more than 70% MMP-9-positive neurons expressed MOR in morphine-treated animals (Figure 3F). In particular, MOR-expressing neurons showed a significant increase in MMP-9 expression after morphine treatment: the percentage of MOR-positive neurons expressing MMP-9 increased from 42.9 ± 10.1% in vehicle-treated group to 74.2 ± 6.0% in morphine-treated group (*P *< 0.05, n = 4) (Figure 3F). Size frequency analysis revealed that majority of MMP-9 and MOR-positive cells were distributed in small- and medium-sized neurons; with mean cross sectional areas of 510 ± 283 μm^2 ^and 407 ± 221 μm^2^, respectively (Figure 4A, B). No significant size shift of the MMP-9 or MOR positive-neurons was detected after morphine treatment (Figure 4A, B).

### MOR agonists increase MMP-9 expression in dissociated DRG neurons

**Figure 5:**
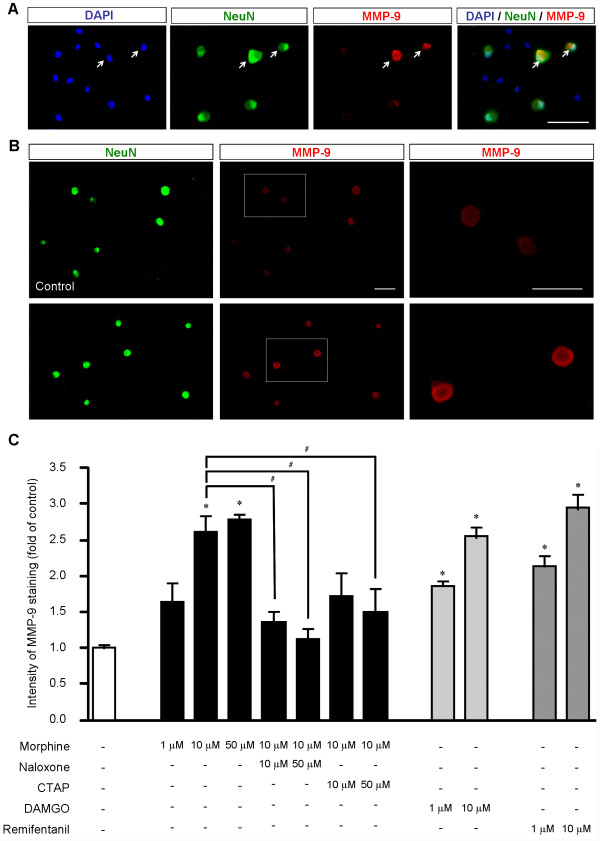
**MOR agonists increase MMP-9 expression in dissociated neurons of DRG cultures**. **(A) **Triple staining of MMP-9, NeuN (neuronal marker), and DAPI (nuclei marker for all cells). Note that MMP-9 is only expressed in neurons (arrows). Scale, 100 μm. **(B) **Double staining showing MMP-9 and NeuN expression in dissociated DRG neurons in control cultures and cultures treated with morphine (10 μM, 2 h). Left panels show all neurons (NeuN+) in the field. Right panels show high magnification images of middle panels. Scales, 100 μm (middle) and 25 μm (right). **(C) **Intensity of MMP-9-positive neurons in DRG cultures after treatment of morphine (1, 10, and 50 μM, 2 h), DAMGO and remifentanil (1 and 10 μM, 2 h), together with the opioid receptor antagonist naloxone and MOR antagonist CTAT (10 and 50 μM). Note that morphine-induced MMP-9 increase is inhibited by naloxone and CTAP. *P < 0.05, compared to control; ^#^*P *< 0.05, ANOVA followed by Bonferroni post hoc test. The data were also analyzed by non-parametric Kruskal-Wallis test followed by Mann-Whitney test. n = 4 cultures.

How does morphine induce MMP-9 expression? Subcutaneous morphine could induce MMP-9 expression in DRG neurons via opioid receptor-independent mechanisms, such as toll-like receptor-mediated activation of glial cells, leading to release of glial mediators to interact with neurons [32,33]. To exclude this possibility, we investigated whether morphine would induce MMP-9 expression through a direct activation of MOR in cultured DRG neurons. As in DRG sections, MMP-9 was also expressed by neurons (NeuN+) in DRG cultures (Figure 5A). Incubation of dissociated DRG neurons with morphine (1 and 10 μM, 2 h) elicited a dose-dependent increase in MMP-9 levels (Figure 5B, C). This increase was not significant (*P *> 0.05, n = 4 cultures) after 1 μM morphine treatment (Figure 5C). However, the intensity of MMP-9 immunoreactivity in DRG cultures significantly increased after 10 μM morphine treatment (2.6 fold of control, *P *< 0.05, n = 4 cultures, Figure 5C). Since 50 μM morphine did not further increase MMP-9 levels, compared to 10 μM morphine (Figure 5C), we used 10 μM morphine for the remaining study. Notably, morphine-evoked MMP-9 increase was found in a ring next to membrane (Figure 5B), indicating a readiness for release of this protease in response to cellular stress. This increase was suppressed by naloxone (10 and 50 μM, *P *< 0.05, n = 4), a general opioid receptor antagonist, and also by D-Phe-Cys-Tyr-D-Trp-Arg-Thr-Pen-Thr-NH_2 _(CTAP, 50 μM, *P *< 0.05, n = 4, Figure 5C), a specific MOR antagonist. In parallel, incubation of DRG cultures with specific MOR agonist DAMGO (1 and 10 μM, 2 h) and remifentanil (1 and 10 μM, 2 h), a short acting MOR agonist [34], also evoked dose-dependent increases in MMP-9 expression (*P *< 0.05, n = 4, Figure 5C). Collectively, our findings support a direct action of opioids on DRG neurons to induce MMP-9 expression, via specific activation of MOR.

### MMP-9 masks opioid analgesia induced by subcutaneous morphine and DAMGO

**Figure 6:**
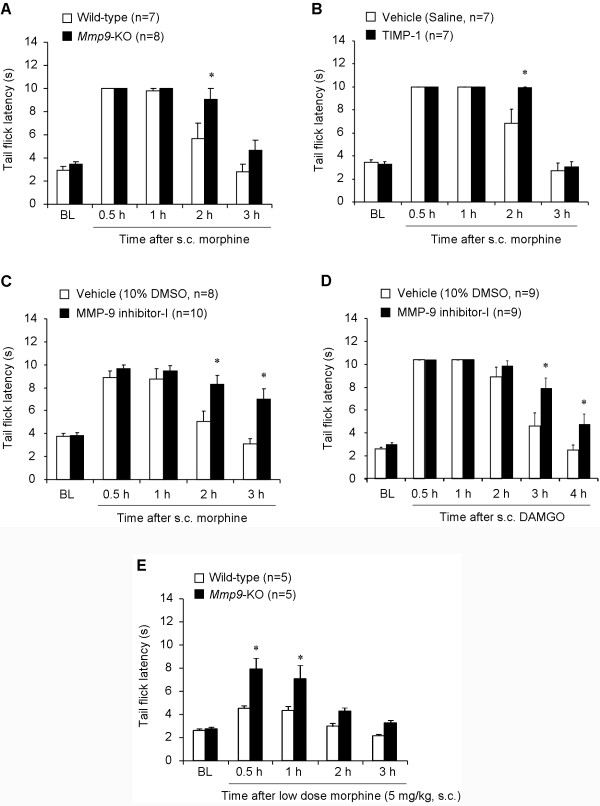
**MMP-9 deficiency or inhibition causes enhanced opioid analgesia after subcutaneous injection of morphine and DAMGO**. **(A) **Morphine (10 mg/kg, s.c) analgesia, as measured by tail flick latency, in wild-type and *Mmp9*-KO mice. *P < 0.05, compared to wild-type control, Student's t-test, n = 7. **(B, C) **Morphine (10 mg/kg, s.c) analgesia in wild-type mice after intrathecal injection of TIMP-1 (0.2 μg, B) and MMP-9 inhibitor-I (0.2 μg, C). *P < 0.05, compared to vehicle (10% DMSO or saline), Student's t-test, n = 7-10 mice. **(D) **DAMGO (10 mg/kg, s.c) analgesia in wild-type mice after intrathecal injection of MMP-9 inhibitor-I (0.2 μg) or vehicle (10% DMSO). *P < 0.05, compared to vehicle, Student's t-test, n = 9 mice. **(E) **Analgesia induced by low dose morphine (5 mg/kg, s.c) in wild-type and *Mmp9*-KO mice. *P < 0.05, compared to Wild-type control. Student's t-test, n = 5 mice. BL, baseline. Note that opioid analgesia by subcutaneous morphine or DAMGO is potentiated and prolonged in *Mmp9*-KOmice and after pharmacological inhibition of MMP-9.

To assess the role of MMP-9 in acute morphine analgesia, we tested morphine analgesia in knockout (KO) mice lacking *Mmp9 *(*Mmp9 *^-/-^) and the same FVB genetic background wild-type (WT) control mice. We did not find difference in the baseline tail flick withdrawal latency between KO and WT mice (Figure 6A, *P *> 0.05, *n *= 7-8 mice). Subcutaneous injection of morphine (10 mg/kg) induced a transient analgesia in WT mice, peaking at 0.5 and 1 h and declining at 2 h. Interestingly, *Mmp9*-KO mice exhibited enhanced morphine analgesia at 2 h (Figure 6A, *P *< 0.05, *n *= 7 mice).

Since *Mmp9*-KO mice are not conditional (see Methods), MMP-9 could regulate opioid analgesia at different sites/levels of the nervous system (e.g., DRG, spinal cord, and supraspinal). To focus on DRG and spinal cord levels, we delivered MMP-9 inhibitors [30], via intrathecal route, to target MMP-9 in the DRG and spinal cord [30]. Intrathecal injection of TIMP-1 (0.1 μg), an endogenous inhibitor of MMP-9 [30], significantly potentiated morphine analgesia at 2 h (Figure 6B, *P *< 0.05, *n *= 7 mice). Intrathecal injection of MMP-9 inhibitor-I (0.2 μg), a small molecule inhibitor of MMP-9 [30], not only potentiated morphine analgesia at 2 h but also prolonged morphine analgesia for > 3 h (Figure 6C, *P *< 0.05, *n *= 8- 10 mice).

Next, we investigated the role of MMP-9 in opioid analgesia induced by a selective MOR agonist, DAMGO. Subcutaneous injection of DAMGO (10 mg/kg) induced analgesia for > 2 h, and this analgesia was potentiated and prolonged by MMP-9 inhibitor-I (i.t., 0.2 μg, Figure 6D, *P *< 0.05, *n *= 9 mice).

Since the dose of 10 mg/kg of morphine induced a ceiling effect in the tail-flick test (near cut-off value at 0.5 and 1 h), reducing the ability to demonstrate an actual enhancement of morphine-induced analgesia in early times, we also tested morphine analgesia at a lower dose (5 mg/kg) in WT and *Mmp9*-KO mice. Of note, morphine analgesia following the lower dose was also significantly potentiated in *Mmp9*-KO mice (Figure 6E, *P *< 0.05, n = 5 mice).

**Figure 7:**
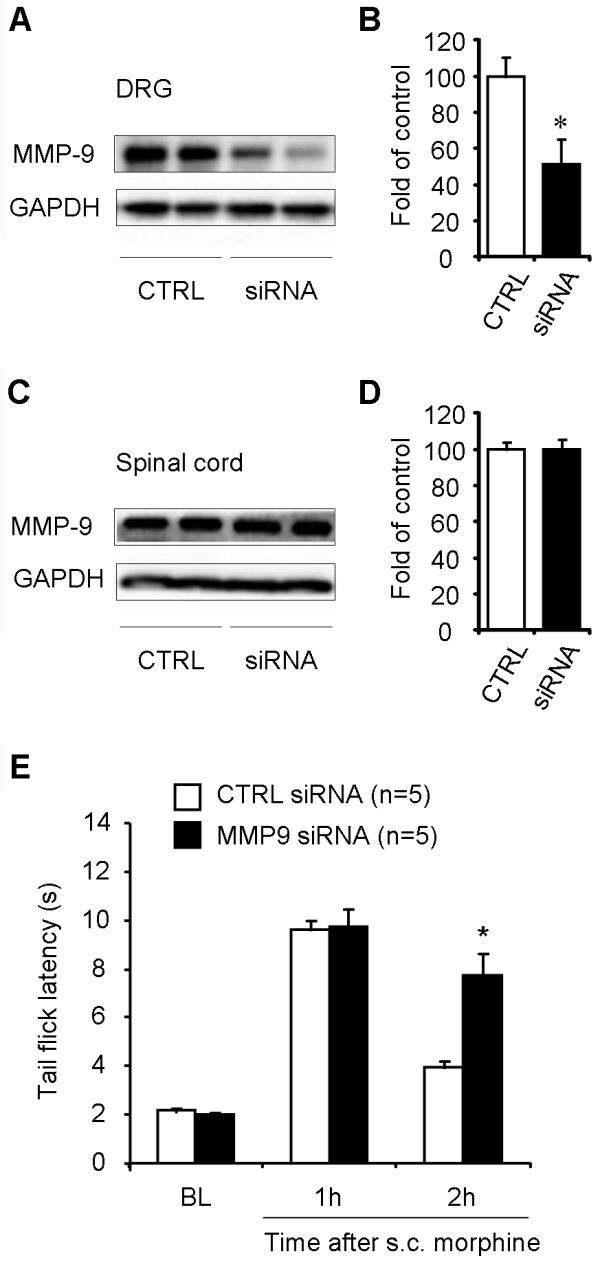
**Intrathecal administration of MMP-9 siRNA reduces MMP-9 expression in DRGs and enhances morphine analgesia**. **(A-D) **Western blotting showing MMP-9 expression in DRGs (A, B) and spinal cord dorsal horns (C, D) after intrathecal injections of MMP-9 siRNA or control siRNA (3 μg, 24 and 2 h before the s.c. morphine injection. B and D are quantification of the MMP-9 bands in DRGs (B) and spinal cords (D). *P < 0.05, compared to control (CTRL); Mann-Whitney test, n = 4 mice. **(E) **Enhanced morphine analgesia after intrathecal treatment of MMP-9 siRNA. *P < 0.05, compared to control siRNA, Student's t-test, n = 5 mice. BL, baseline.

We further evaluated the role of MMP-9 in opioid analgesia using siRNA strategy to knockdown MMP-9 expression in the DRG/spinal cord via intrathecal route [25,35]. Our previous study showed that intrathecal injections of MMP-9 siRNA specifically reduced MMP-9 but not MMP-2 activity in DRGs after nerve injury [30]. The same siRNA treatment (3 μg, i.t., given 24 and 2 h before s.c. morphine injection) also reduced MMP-9 expression in the DRG (Figure 7A, B, *P *< 0.05, *n *= 5 mice) but not in the spinal cord (Figure 7C, D, *P *> 0.05, *n *= 5 mice). One possible explanation for the differential knockdown of DRG vs. spinal cord MMP-9 by intrathecal siRNA treatment is that in the context of our experimental condition the inducible MMP-9 expression in DRGs is more sensitive to siRNA treatment than the constitutive MMP-9 expression in spinal cords, as shown in our previous study [25,30]. Importantly, the MMP-9 siRNA treatment also enhanced morphine analgesia (10 mg/kg) at 2 h (Figure 7E, *P *< 0.05, *n *= 5 mice). Since the siRNA treatment only reduced MMP-9 levels in DRGs but not in spinal cords, MMP-9 expressed by DRG cells should play an important role in mediating pronociceptive actions of opioid.

### Intrathecal morphine and remifentanil induce MMP-9 expression in DRGs

**Figure 8:**
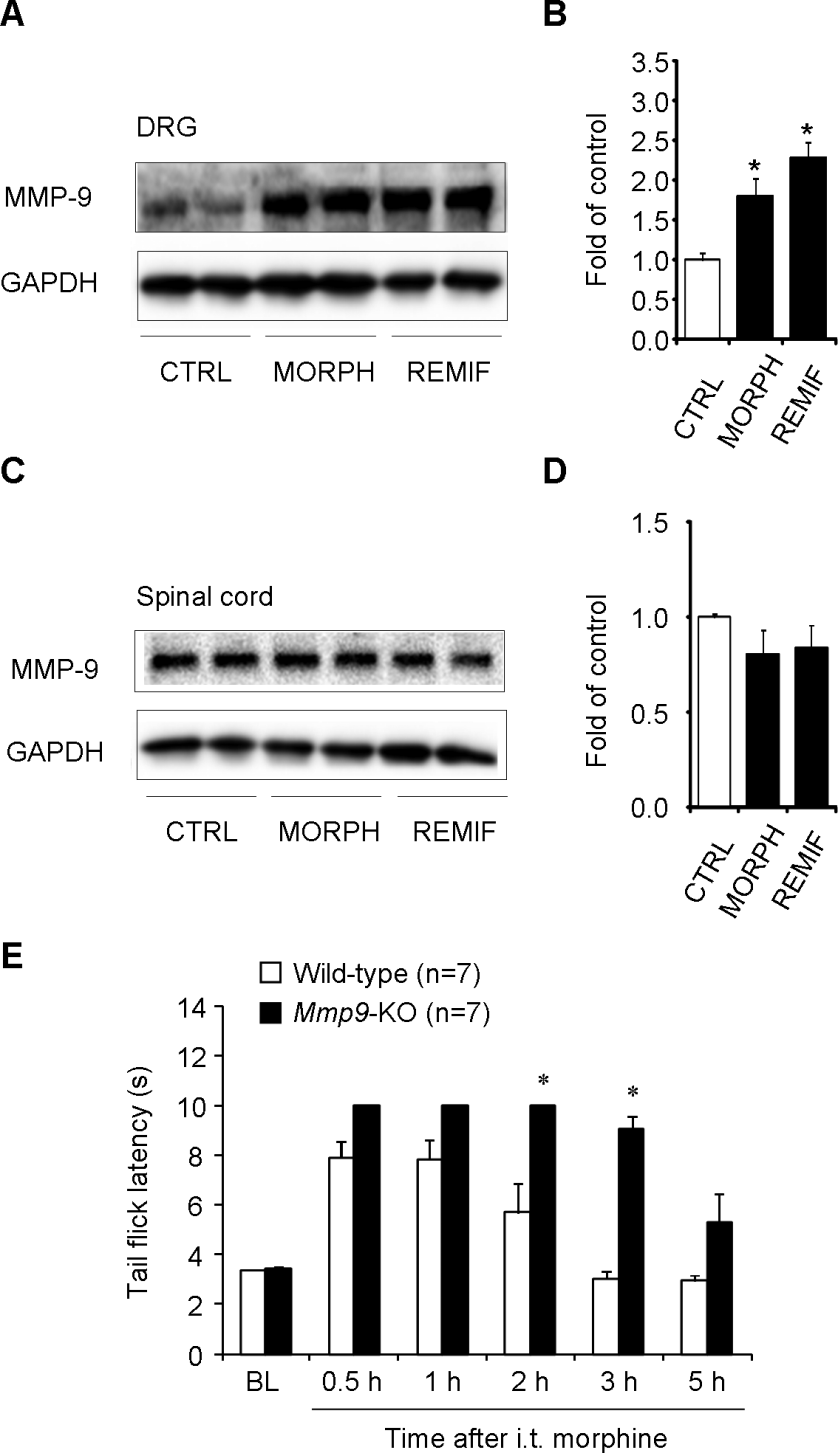
**Up-regulation of MMP-9 expression in DRGs but not spinal cords after intrathecal morphine or remifentanil and potentiation of intrathecal morphine analgesia in *Mmp9 *deficient mice**. **(A-D) **Western blotting showing MMP-9 expression in DRGs (A, B) and spinal cord dorsal horns (C, D) 2 h after intrathecal injection of morphine (10 nmol) or remifentanil (1 nmol). B and D are quantification of the MMP-9 bands in DRGs (B) and spinal cords (D). *P < 0.05, compared to control, ANOVA followed by Bonferroni post hoc test, n = 4 mice. CTRL, control; MORPH, morphine; REMIF, remifentanil. **(E) ***Mmp9*-KO mice exhibit enhanced and prolonged analgesia after intrathecal morphine (10 nmol). *P < 0.05, compared to Wild-type control, Student's t-test, n = 7 mice. BL, baseline.

Our data showed that subcutaneous morphine elicited MMP-9 expression in the DRG but not in the spinal cord (Figure 1). This failure of systemic morphine to induce MMP-9 expression in the spinal cord may result from limited access of morphine to the spinal cord (part of the central nervous system) following systemic injection. To determine whether acute delivery of MOR agonists to the spinal cord would induce MMP-9 expression in the spinal cord, we administrated morphine and remifentanil intrathecally via lumbar puncture and collected DRG and spinal cord dorsal horn tissues 2 h later. Intrathecal morphine (10 nmol) and remifentanil (1 noml) elicited significant MMP-9 expression in DRGs (Figure 8A, B; 1.8 fold and 2.3 fold of control, respectively, *P *< 0.05, n = 4 mice). In contrast, intrathecal morphine and remifentanil failed to induce MMP-9 expression in the spinal cord dorsal horn (Figure 8C, D; *P *> 0.05, n = 4 mice). These results further suggest that DRG is a primary site for acute opioid-induced MMP-9 expression.

We also examined the correlation between intrathecal morphine-induced MMP-9 expression and intrathecal morphine-induced analgesia. Intrathecal morphine (10 nmol) induced robust analgesia in WT mice, which lasted > 2 h and recovered after 3 h (Figure 8E). Compared to WT mice, *Mmp9*-KO mice displayed enhanced analgesia at 2 h and prolonged analgesia for > 3 h (Figure 8E; *P *< 0.05, n = 7 mice).

### MMP-9 does not regulate morphine-induced hyperalgesia

**Figure 9:**
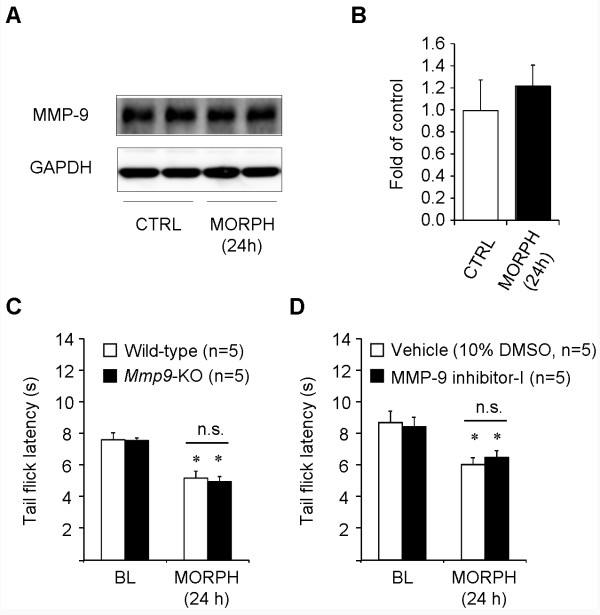
**MMP-9 deficiency does not alter opioid-induced hyperalgesia after subcutaneous morphine**. **(A, B) **Western blot gel (A) and band density (B) showing MMP-9 expression in lumbar DRGs 24 h after morphine (MORPH) injection. *P > 0.05, compared to naive control (CTRL), Student's t-test, n = 4 mice. **(C, D) **Subcutaneous morphine (5 mg/kg) induces comparable heat hyperalgesia in both wild-type and *Mmp9*-KO mice (C) and also in wild-type mice pretreated with intrathecal MMP-9 inhibitor-I (0.2 μg) or vehicle (10% DMSO). *P < 0.05, compared to corresponding baseline (BL), Student's t-test, n.s., no significance. n = 5 mice. Morphine-induced heat hyperalgesia was tested by tail flick latency in 48°C hot water.

Finally, we tested if morphine could induce MMP-9 expression in a late-time point (24 h) and its relevance to OIH. Subcutaneous morphine only induced a transient MMP-9 expression in the first 3 h (Figure 1) but not after 24 h (Figure 9A, B, *P *> 0.05, n = 4 mice). In order to unmask OIH, we tested tail flick latency at 48°C instead of 52°C, at 24 h after morphine injection, according to the protocol of Elhabazi et al. [15]. Subcutaneous morphine induced a significant heat hyperalgesia at 24 h in WT mice (Figure 9C, *P *< 0.05, n = 5 mice). Notably, OIH is comparable in WT and *Mmp9*-KO mice (Figure 9C, *P *> 0.05, n = 5 mice). In parallel, intrathecal pre-treatment of MMP-9 inhibitor-I also failed to prevent OIH at 24 h (Figure 9D, *P *> 0.05, n = 5 mice). Thus, MMP-9 is only involved in masking opioid-induced analgesia in the first several hours but not participated in OIH after 24 h.

## Discussion

Our data demonstrated that acute morphine treatment induced rapid MMP-9 up-regulation in primary sensory neurons to counteract opioid-induced analgesia but had no role in promoting OIH. First, subcutaneous and intrathecal morphine induced a rapid and transient (< 3 h) MMP-9 up-regulation in DRG neurons but not in the spinal cord. Second, MOR agonists morphine, DAMGO, and remifentanil increased MMP-9 expression in dissociated DRG neurons, in a MOR-dependent manner. Third, *Mmp9 *deletion or inhibition (via intrathecal route) enhanced and prolonged opioid analgesia in the first several hours but did not impact OIH after 24 h. To our knowledge, this is the first time to demonstrate that different molecular mechanisms control different pronociceptive actions of opioids: opioid-induced anti-analgesia during acute opioid analgesia (first 3 h) vs. OIH after opioid withdrawal (> 24 h).

Several lines of evidence from preclinical and clinical studies suggest that opioids may produce paradoxical hyperalgesia [17]. OIH is thought to result from the up-regulation of pronociceptive pathways within the central and peripheral nervous systems [18,16]. Traditionally, OIH has been associated with chronic pain and analgesic tolerance to opioids after long-term exposure to opioids. It is well documented that chronic opioid induces the expression of pronociceptive genes in DRGs (e.g., TRPV1, substance P, CGRP, chemokine) [26,27,36] and spinal cords (e.g., prodynorphin, NK-1, and PKCγ) [37-39], promoting opioid-induced antinociceptive tolerance and hyperalgesia. Sustained morphine exposure also induces MMP-9 up-regulation in the spinal cord, leading to opioid-induced withdrawal responses that might be associated with morphine dependence [31]. Furthermore, recent studies suggest that acute OIH can occur after intraoperative or postoperative administration of high doses of potent opioids, leading to increased postoperative pain [17,40]. For example, enhancement of experimentally induced hyperalgesia was observed in humans after infusion of remifentanil, a short-acting potent opioid, in several volunteer studies [40]. In rodents, acute fentanyl not only produces potent analgesia but also elicits secondary long-lasting hyperalgesia for several days [19]. A single dose of intrathecal morphine induces long-lasting hyperalgesia in rats [20]. Activation of spinal cord NMDA receptors has been strongly implicated in the development of OIH, since the use of clinically available NMDA receptor antagonists such as ketamine and dextromethorphan can attenuate OIH [17,20,40]. Recently, Drdla et al. (2009) have shown that abrupt opioid withdrawal induces LTP in spinal cord dorsal horn neurons *via *activation of opioid receptors and NMDA receptors, providing a cellular mechanism of OIH [22]. Zhou et al. (2010) have further demonstrated that DAMGO-induced spinal LTP requires TRPV1-expressing primary afferents, suggesting an involvement of presynaptic mechanisms [24]. Despite the progress in revealing mechanisms of OIH, it is still unclear whether acute opioid can induce gene expression in primary sensory neurons to mediate the pronociceptive actions of opioid.

We demonstrated a novel role of MMP-9 in masking opioid analgesia. First, morphine analgesia induced by both subcutaneous and intrathecal morphine was potentiated and prolonged in *Mmp9*-KO mice. Second, intrathecal injection of two different MMP-9 inhibitors, MMP-9 inhibitor-1, a small molecule inhibitor, and TIMP-1, a peptide inhibitor, potentiated and prolonged analgesia by subcutaneous morphine and DAMGO analgesia (Figure 6). Increasing evidence demonstrates that intrathecal route can target both DRG and spinal cord cells. Agents with different chemical properties have been shown to affect DRG cells via intrathecal route, including small molecules such as MAP kinase and MMP-9 inhibitors [30,41] and large molecules such as growth factors and peptides [42,43], as well as antisense oligodeoxynucleotides [44,45] and siRNAs [25,46]. Intrathecal p38 inhibitor rapidly (< 30 min) inhibited p38 activation in DRG neurons [47]. Intrathecal MMP-9 also causes IL-1β cleavage in the DRG [30]. Notably, intrathecal morphine and remifentanil induced robust MMP-9 upregulation in DRG (Figure 8).

In parallel with a role of MMP-9 in masking acute opioid analgesia, we found MMP-9 up-regulation in DRGs but not spinal cords after subcutaneous morphine (Figure 1) and intrathecal morphine or remifentanil (Figure 8), suggesting that MMP-9-evoked pronociceptive actions after acute opioid are likely to be mediated by MMP-9 from DRG. Gelatin zymography data further indicated a correlated increase in MMP-9 activity in DRG following acute morphine. However, we did not observe any increase in MMP-2 activity after acute morphine, in support of our previous report that MMP-2 is not involved in early-phase development of neuropathic pain [30]. We also found a corresponding increase in MMP-9 mRNA levels in DRG neurons following acute morphine treatment (Figure 2), suggesting a possible transcription regulation of MMP-9. However, the increases of morphine-induced MMP-9 protein and activity occurred prior to the MMP-9 mRNA increase (Figures 1 and 2), suggesting possible translational regulation (protein increase in the absence of mRNA increase) and post-translational regulation (increase in MMP-9 activity in the absence of protein synthesis), in addition to transcriptional regulation. Of note, we found strong mRNA signals in subcellular regions close to the surface of DRG neurons (Figure 2D, E). Recent evidence also demonstrated translocation of RNA granules in living neurons [48] and RNA translocation was found in DRG neurons in chronic pain [49]. Consistently, strong MMP-9 immunoreactivity was found in a ring close to the surface of cultured DRG neurons (Figure 5B). Although the finding of morphine-induced MMP-9 mRNA trafficking is interesting, the functional significance of the mRNA translocation remains to be investigated.

Our data indicated that activation of MOR was required for morphine-induced MMP-9 expression in DRG neurons. (i) MMP-9 increase was enriched in MOR-positive neurons following subcutaneous morphine (Figure 3). (ii) Morphine-induced MMP-9 expression in dissociated DRG neurons was suppressed by naloxone and the selective MOR antagonist CTAP (Figure 5C). (iii) MOR agonists DAMGO and remifentanil increased MMP-9 expression in dissociated neurons (Figure 5C). The signaling mechanisms underlying opioid-induced MMP-9 up-regulation in primary sensory neurons are still elusive. Activation of MAP kinase pathways has been implicated in chronic morphine-induced gene expression in DRG neurons [50,51]. It is of interest to test whether MAP kinase pathways are also involved in acute morphine-induced MMP-9 expression. Although our results support a MOR-dependent mechanism of MMP-9 induction, morphine may also induce gene expression via MOR-independent mechanisms. Intrathecal injection of morphine-3-glucuronide, a major morphine metabolite, has been shown to enhance pain via toll-like receptor 4 (TLR4) [32].

Our data also suggested that MMP-9 at DRG level plays a major role in sabotaging opioid analgesia, because (i) subcutaneous and intrathecal MOR agonists induced MMP-9 at the DRG but not spinal cord level and (ii) intrathecal MMP-9 inhibitors and siRNA potentiated and prolonged opioid analgesia. In particular, low-dose morphine-induced analgesia was even potentiated in the acute phase (0.5 and 1 h) in *Mmp9*-KO mice (Figure 6E). How can MMP-9 up-regulation in DRG neurons by morphine induce pronociceptive actions? Our previous study showed that MMP-9 could be released in DRG cultures in an activity-dependent manner [30], which may cause active cleavage of IL-1β [28]. IL-1β is activated via cleavage from its precursor by caspase-1 (the IL-1β converting enzyme), as well as by other enzymes such as trypsin, elastase, collagenase, and cathepsin G, MMP-9, and MMP-2 [29,52,53]. IL-1β activation in DRGs is increased after intrathecal MMP-9 treatment [31]. Further, MMP-9-induced pain hypersensitivity is abrogated by IL-1β blockade, suggesting that IL-1β activation is a downstream mechanism underlying MMP-9-induced pronociceptive actions [30]. In DRGs, IL-1β is expressed by satellite glial cells [30,54,55] and neurons [30,56]. DRG neurons and nociceptors also express IL-1 receptors [57]. Accumulating evidence suggests that IL-1β causes hyperexcitability of sensory neurons by increasing sodium currents and suppressing potassium currents [55,57,58]. Intrathecal administration of interleukin-1 receptor antagonist can potentiate both intrathecal and systemic morphine-induced analgesia [59,60]. In a recent study, we have demonstrated that MMP-9 is required for acute morphine-induced activation of IL-1β and satellite glial cells in DRGs via neuron-glial interactions [61].

Thus, it is conceivable that MMP-9 might antagonize morphine analgesia via IL-1β signaling, although we should not exclude the possibility that MMP-9 may regulate morphine analgesia via cleavage of other signaling molecules such as TNF-α and chemokines [28].

In summary, opioids are still the most effective analgesics for the treatment of moderate to severe pain. Apart from well-known inhibitory effects of acute opioids, acute opioids also elicit paradoxical excitatory and pronociceptive actions in the spinal cord via presynaptic [24] and postsynaptic [21,22] mechanisms. We further postulate that opioid-induced excitatory and pronociceptive effects are sequential: (1) anti-analgesic effect during acute opioid analgesia (first 3 h), (2) hyperalgesic effect after opioid withdrawal (after 24 h), and (3) antinociceptive tolerance (after several days). We also postulate that these pronociceptive effects of opioids could be mediated by distinct mechanisms. Our data suggest that acute opioid treatment, via either subcutaneous or intrathecal route, can elicit transient MMP-9 induction in primary sensory neurons to antagonize/mask acute morphine analgesia in the first several hours. However, MMP-9 is not involved in OIH after 24 h. Thus, targeting MMP-9 may enhance and prolong opioid analgesia in pain conditions such as acute postoperative pain.

## Methods

### Animals

All experiments were performed in accordance with the guidelines of the National Institutes of Health and the International Association for the Study of Pain. All animals were used under Harvard Medical School Animal Care institutional guidelines. Adult male mice (25-35 g) were used for behavioral and biochemical studies. These mice included CD1 mice (Charles River Laboratories), *Mmp9 *knockout (*Mmp9***^-/-^**) mice with FVB background, and FVB control wild-type mice. *Mmp9 *knockout mice and FVB wild-type mice were obtained from Jackson Laboratories and bred at Thorn Research Building Animal Facility of Harvard Medical School. As described on the website of Jackson laboratories, a targeting vector containing a neomycin resistance gene driven by the mouse phosphoglycerate kinase promoter was used to disrupt most of exon 1 and all of intron 2 of the *Mmp9 *gene. The construct was electroporated into 129S-derived ZW4 embryonic stem (ES) cells. Correctly targeted ES cells were injected into C57BL/6 J blastocysts. The mice were maintained in FVB background for more than 5 generations. Mice that are homozygous null for the *Mmp9 *gene were used in this study. They are viable and fertile and do not show difference in overall weight and behavior compared with wild-type mice [30]. Animals were housed in a 12 h light/dark room with access to food and water *ad libitum*.

### Drugs and administration

We purchased morphine sulphate and remifentanil from Hospira Inc, MMP-9 inhibitor-I from Calbiochem (Catalogue # 444278), D-Ala(2), N-Me-Phe(4), Gly-ol(5))-enkephalin (DAMGO), naloxone, and CTAP (D-Phe-Cys-Tyr-D-Trp-Arg-Thr-Pen-Thr-NH_2_) from Sigma, and tissue inhibitor of MMP-I (TIMP-1) from R & D. Morphine, DAMGO, and remifentanil were freshly prepared in saline and administered subcutaneously [5 or 10 mg/kg for morphine and DAMGO, or intrathecally (10 nmol for morphine and 1 nmol for remifentanil)]. MMP-9 inhibitor-I (0.2 μg) and TIMP-1 (0.1 μg) were dissolved in 10% DMSO and saline, respectively. The inhibitors and doses of MMP-9 were chosen based on our previous studies [30]. The MMP-9 inhibitors were intrathecally injected (0.2 μg in10 μl) 30 min before the morphine injection. The MMP-9 and control siRNAs were purchased from Dharmacon and the sequences of these siRNAs were described in our previous study [30]. siRNA was dissolved in RNase-free water at the concentration of 1 μg/μl as stock solution, and mixed with polyethyleneimine (PEI, Fermentas), 10 min before injection, to increase cell membrane penetration and reduce the degradation [30,35]. PEI was dissolved in 5% glucose, and 1 μg of siRNA was mixed with 0.18 μl of PEI. siRNA (3 μg) was intrathecally injected 24 and 2 h before the morphine injection. For intrathecal injection, a lumbar puncture was made at L5-L6 level with a 30 gauge needle under a brief isoflurane anesthesia [62].

### Behavioral test

Morphine analgesia was evaluated by tail-flick latencies in hot water [63]. Briefly, tail-flick test was performed by gently holding the mouse wrapped with a terry towel and kept tail exposed. Then one third of the length of the tail was immersed into the 52°C hot water, and the response latency was recorded at the removal of the whole tail from the water. A maximum cut-off value of 10 s was set to avoid thermal injury. To investigate opioid-induced hyperalgesia, we also measured tail flick latency at 48°C, as described in a recent study [64]. The experimenters were blinded to the treatment or genotype.

### Immunohistochemistry

Two hours after morphine or vehicle (saline) injection, animals were terminally anesthetized with isoflurane and perfused through the ascending aorta with PBS followed by 4% paraformaldehyde with 1.5% picric acid in 0.16 M PB, and the DRGs (L4/L5) were removed and postfixed in the same fixative overnight. DRG sections (12 μm) were cut in a cryostat and processed for immunofluorescence. All the sections were blocked with 10% goat serum, and incubated over night at 4°C with the primary antibodies against MMP-9 (rabbit, 1:500-2000, Chemicon) and MOR (guinea pig, 1:1000, Chemicon), with 5% goat serum. The sections were then incubated for 1 h at room temperature with Cy3- or FITC- conjugated secondary antibody (1:400, Jackson Immunolab) with 1% goat serum. For double immunofluorescence, sections were incubated with a mixture of polyclonal and monoclonal primary antibodies followed by a mixture of FITC- and CY3-congugated secondary antibodies [65]. The stained sections were examined under a Nikon fluorescence microscopy, and images were captured with a CCD camera (SPOT, Diagnostic Instruments). All images were analyzed with NIH Image software or Adobe Photoshop.

### Western blot

Animals were terminally anesthetized with isoflurane at 1, 2, 3, and 24 h after morphine injection and transcardially perfused with PBS. DRGs (L4/L5) and spinal cord dorsal horn segments (L4-L5) were rapidly removed and homogenized in a lysis buffer containing a cocktail of protease inhibitors and phosphatase inhibitors [66]. The protein concentrations were determined by BCA Protein Assay (Pierce). Thirty micrograms of proteins were loaded into each lane and separated on SDS-PAGE gel (4-15%, Bio-Rad). After the transfer, the blots were incubated overnight at 4°C with polyclonal antibody against MMP-9 (rabbit, 1:1000, Chemicon). For loading control, the blots were probed with β-tubulin or GAPDH antibody (rabbit, 1:5000, Sigma).

### Gelatin zymography

Because MMP-2 and MMP-9 are gelatinases, we used gelatin zymography to determine the activity of MMP-9 and MMP-2 [28]. Animals were terminally anesthetized with isoflurane at 0.5, 1, 2, and 3 h after morphine injection and transcardially perfused with PBS. DRGs (L4/L5) were rapidly removed and homogenized in the same lysis buffer as we used for Western blotting. Gelatin zymography was conducted as previously described [30,67]. Ten micrograms of proteins were loaded per lane into the wells of precast gels (10% polyacrylamide minigels containing 0.1% gelatin, Novex). After electrophoresis, each gel was incubated with 100 ml of development buffer (50 mM Tris base, 40 mM HCl, 200 mM NaCl, 5 mM CaCl2, and 0.2% Brij 35, Novex) at 37°C for 14-18 h. Staining was performed with 100 ml of 0.5% Coomassie blue G-250 in 30% methanol and 10% acetic acid for 1 h. Gelatinolytic activity was demonstrated as clear zones or bands.

### Primary DRG cultures and immunocytochemistry

DRGs were removed aseptically from 4-week old mice and first incubated with collagenase (1.25 mg/ml, Roche)/dispase-II (2.4 units/ml, Roche) at 37°C for 90 min, then digested with 0.25% trypsin (Cellgro) for 8 min at 37°C, followed by 0.25% trypsin inhibitor (Sigma). Cells were then mechanically dissociated with a flame polished Pasteur pipette in the presence of 0.05% DNAse I (Sigma). Dissociated DRG neurons were cultured in neurobasal defined medium with 2% B27 supplement (Invitrogen) on coverslips precoated with poly-D-lysine (100 μg/ml, Sigma) and laminin (10 μg/ml, Invitrogen) at 36.5°C, with 5% carbon dioxide, in the presence of 5 μM AraC to inhibit the proliferation of non-neuronal cells,. To remove myelin debris and cell clumps, a partial purification step was performed by centrifugation through a BSA cushion (15%, Sigma). DRG neurons were grown in cultures for 24 h before use. DRG neurons were incubated with morphine (1, 10 and 50 μM), DAMGO (1 and 10 μM), and remifentanil (1 and 10 μM), as well as morphine plus naloxone (10 and 50 μM), and morphine plus CTAP (D-Phe-Cys-Tyr-D-Trp-Arg-Thr-Pen-Thr-NH_2_) (10 and 50 μM) for 2 h. The concentrations of opioids were based on previous studies in primary DRG cultures [39,40]. For immunocytochemistry, DRG neurons were fixed with 4% paraformaldehyde for 20 min and incubated with MMP-9 (1:1000, rabbit, Chemicon) and NeuN (1:1000, mouse, Millipore) overnight at 4°C. DAPI (4',6-diamidino-2-phenylindole, 1:5000, Invitrogen) was used to stain the cell nucleus.

### Quantitative real-time PCR

Mice were sacrificed after terminal anesthesia with isoflurane, and the L4-L5 DRGs were rapidly dissected. Total RNA was extracted using RNeasy Plus Mini kit (Qiagen). Quantity and quality of the eluted RNA samples were verified by NanoDrop spectrophotometer (Thermo Fisher Scientific). A total of 0.5 μg of RNA was reverse transcribed using Omniscript reverse transcriptase according to the protocol of the manufacturer (Qiagen). Specific primers for MMP-9 and TIMP-1 as well as GAPDH control were designed using IDT SciTools Real-Time PCR software (Integrated DNA Technologies). The sequences of these primers were described in Table 1. Quantitative PCR amplification reactions contained the same amount of RT product: 7.5 μl of 2 × iQSYBR-green mix (BioRad) and 300 nM of forward and reverse primers in a final volume of 15 μl. The thermal cycling conditions comprised 3 min polymerase activation at 95°C, 45 cycles of 10 s denaturation at 95°C and 30 s annealing and extension at 60°C, followed by a DNA melting curve for the determination of amplicon specificity. All experiments were performed in duplicate. Primer efficiency was obtained from the standard curve and integrated for calculation of the relative gene expression, which was based on real-time PCR threshold values of different transcripts and groups [68,69].

**Table 1:** The sequences of the primers for RT-PCR

Target gene	Forward primers	Reverse primers	**Genbank No**.
MMP-9	GATCCCCAGAGCGTCATTC	CCACCTTGTTCACCTCATTTTG	NM013599
TIMP-1	CTCAAAGACCTATAGTGCTGGC	CAAAGTGACGGCTCTGGTAG	NM011593
GAPDH	TCCATGACAACTTTGGCATTG	CAGTCTTCTGGGTGGCAGTGA	XM001473623

### In situ hybridization

Animals were transcardially perfused with PBS and 4% paraformaldehyde after terminal anesthesia with isoflurane. DRGs were collected and post-fixed overnight. DRG tissues were sectioned at a thickness of 12 μm in a cryostat and mounted on superfrost plus slides. MMP-9 riboprobes (0.47 kb) was generated by PCR. The reverse primer contains T7 RNA polymerase binding sequence (TGTAATACGACTCACTATAGGGCG) for the generation of the antisense riboprobe. DNA sequences were transcribed *in vitro *with T7 RNA polymerase (Promega) in the presence of digoxigenin-labeling mix. DRG sections were hybridized with MMP-9 riboprobe (1 μg/ml) overnight at 65°C. After washing, sections were blocked with 20% serum for 1 h at room temperature followed by incubation with alkaline phosphatase-conjugated anti-digoxigenin antibody (1:2000; Roche Diagnostics) overnight at 4°C. Sections were then incubated with a mixture of nitro-blue tetrazolium (NBT) and 5-bromo-4-chloro-3-indolyl-phosphate (BCIP) in alkaline phosphatase buffer for 24-48 h for color development. *In situ *images were captured with a Nikon microscope under bright-field.

### Quantification

For cell counting in DRG sections, we randomly selected 4 sections from each DRG/animal and counted i) total number of cells and ii) number of mRNA-positive (*in situ *hybridization) or immunoreactive cells in these 4 sections and calculated the % of positive cells in DRG sections, as we previously reported [1,65]. We included 4 animals per group for the quantification of histochemistry in DRG tissue sections. To quantify MMP-9 staining in cultured DRG neurons, we measured the intensity of MMP-9-positive neurons as we previously reported [41,70]. We included 4 cultures per condition and examined all the cells in each culture. The total number of cells in each culture condition varied from 399 to 746. Cell counting was conducted in a blinded manner. For Western blotting and gelatin zymography, we measured the intensities of positive bands and included 4 animals per group for biochemical analyses. For behavioral tests, we included n = 5-10 animals per group.

### Statistical analysis

All the data are expressed by mean ± SEM. Differences between groups were tested using ANOVA followed by posthoc Bonferroni test and Student's t-test. Some data (n = 4 samples) were also tested by non-parametric Kruskal-Wallis test and Mann-Whitney test, and the same statistical results were obtained. The criterion for statistical significance was *P *< 0.05.

## Competing interests

The authors declare that they have no competing interests.

## Authors' contributions

YCL and TL performed behavioral tests; TB and TL performed biochemical and histochemical experiments; PHT designed siRNA sequences; TB, TL, YCL, and RRJ designed the experiments; and RRJ and TB wrote the manuscript. All authors read and approved the final manuscript.

## Supplementary Material

Additional file 1 **Supplemental Figures**. Click here for file
