# Supplementary material for: Acute morphine induces matrix metalloproteinase-9 up-regulation in primary sensory neurons to mask opioid-induced analgesia in mice
Source: Mol Pain. 2012 Mar 25;8:19. doi: 10.1186/1744-8069-8-19 (PMC3353172; doi:10.1186/1744-8069-8-19)
Supplement: Additional file 1 — Supplemental Figures. [file 1744-8069-8-19-S1.PDF]

Acute morphine induces matrix metalloproteinase-9 up-regulation in primary sensory neurons to mask opioid-induced analgesia in mice

Yen-Chin Liu, Temugin Berta, Tong Liu, Ping-Heng Tan, and Ru-Rong Ji

Supplemental Figures (1-3)

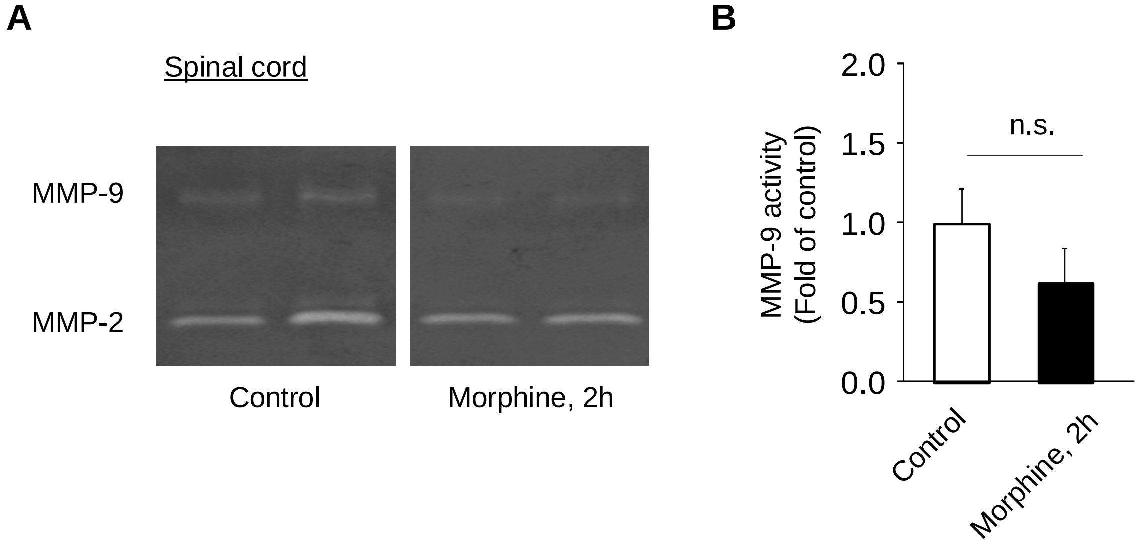

<u>Supplemental Figure 1</u>. Subcutaneous morphine does not increase MMP-9 activity in the spinal cord. (A) Gelatin zymography showing the MMP-9 and MMP-2 activity in the lumbar spinal cord dorsal horn 2 h after morphine (s.c., 10 mg/kg). **(B)** Quantification of the intensity of MMP-9 bands. P>0.05, Student's t-test, n=4 mice. n.s. no significance.

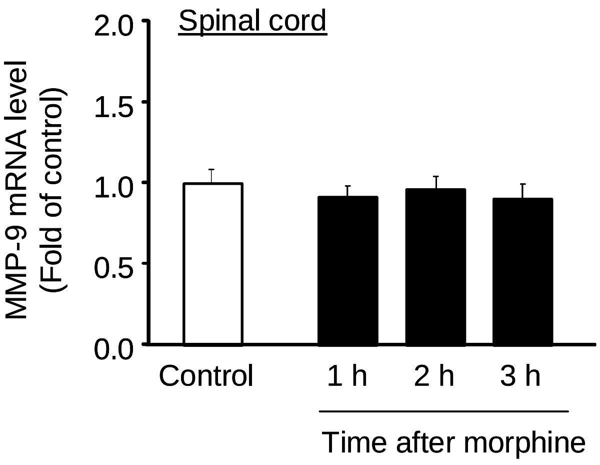

Supplemental Figure 2. RT-PCR showing that subcutaneous morphine does not change MMP-9 mRNA expression in the spinal cord dorsal horn. $P>0.05$, control vs. different time point, ANOVA followed by Bonferroni post hoc test, n=4 mice.

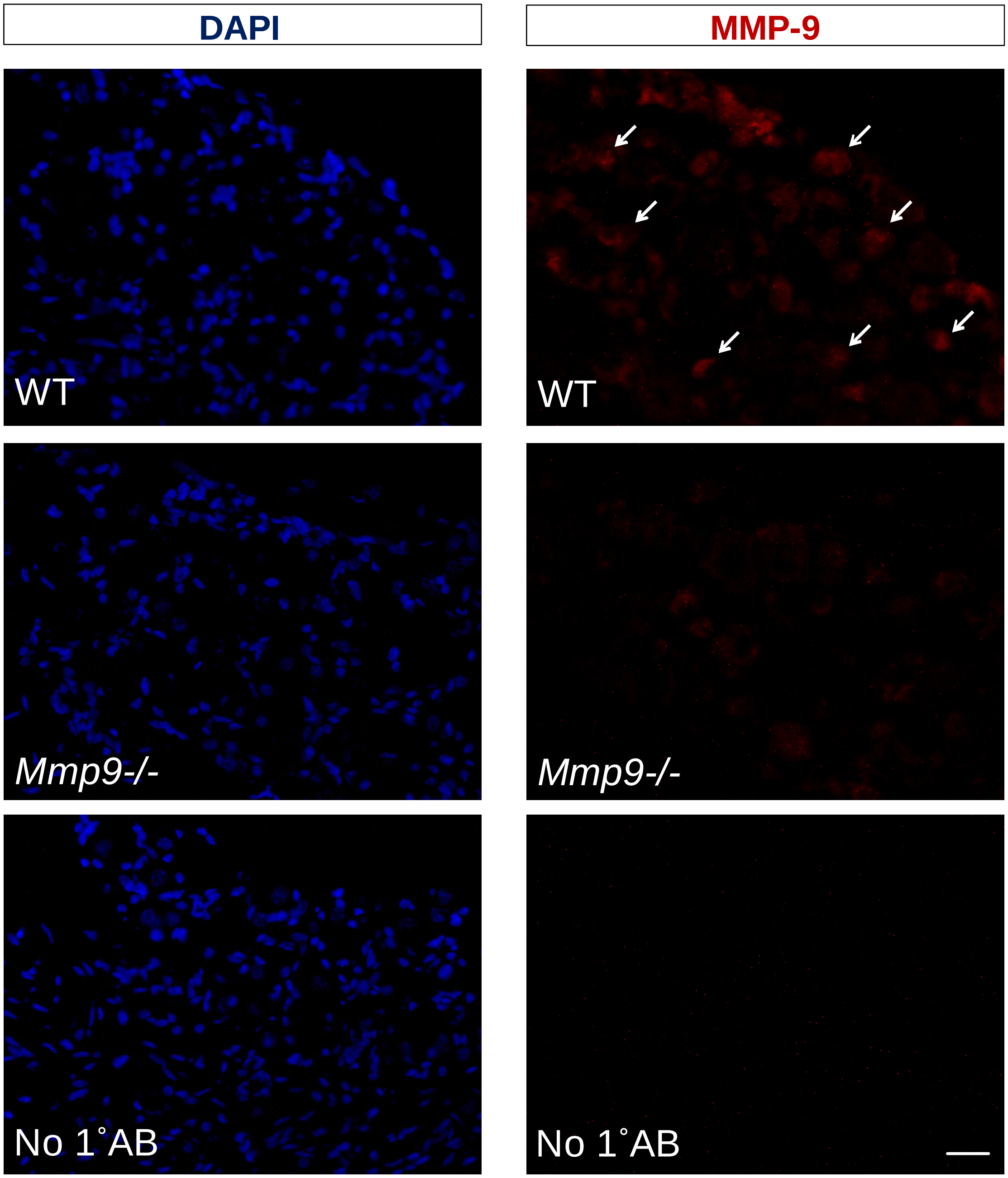

<u>Supplemental Figure 3</u>. MMP-9 immunostaining in DRG sections of WT and *Mmp9* KO mice. MMP-9 immunoreactivity was found in DRG neurons (arrows) of WT mice but not in DRG sections of *Mmp9* KO mice. MMP-9 immunoreactivity was also lost in the absence of the primary antibody (lower panel). The dilution of MMP-9 antibody is 1:2000. Double staining with DAPI shows morphology of the same DRG sections for MMP-9 staining. Scale, 50 μm.
